# The "Dreadnought" Hospital. A Successful and Busy Year

**Published:** 1916-03-18

**Authors:** 


### THE "DREADNOUGHT" HOSPITAL.

### A Successful and Busy Year.

At the annual Court of Governors of this Society, held at the Princes Restaurant, Piccadilly, W., on Tuesday, March 14, Sir Perceval Nairne, in moving the adoption of the annual report, said that during the year 536 patients were received from both the merchant service and the Royal Navy, and the hospital's capacity was taxed to its utmost in the last three months of 1915, 320 patients being in the wards at the same time. Despite the greatly increased cost of food, drugs, and appliances, the annual subscriptions had, however, increased sufficiently to keep the balance on the right side of the account. In recognition of Lord Devonport's great share in the success of the special appeal, the Court had decided to name a floor of the institution "The Devonport Floor."

The Chairman (Major-General Lessard, C.B., Canadian Expeditionary Force) said that the appeal for contributions issued throughout Canada had resulted in £4,000 being raised in Toronto alone. He expected proportionately good results from other cities. He had written to every Mayor, and the Press of the Dominion had given the movement cordial support.

The Right Hon. Lord Devonport, in proposing the election of the officers of the Society for the ensuing year, referred to the warm response to the special appeal from financial, shipping, and industrial firms, a great impetus doubtless being given by the contributions of the King, Queen Alexandra, and the Prince of Wales. Mr. Jones, Mr. Sturge, and Mr. Devett had been particularly successful as collectors, and he could not speak sufficiently highly of the fine response of Canada and the other Dominions. Ten days after the appeal was launched £10,000 had been received, in a month it was twice that sum, and he fully expected the total would be about £50,000. The costs incidental to the work were only about 3 per cent., as against the usual 5 per cent. reckoned by collecting agencies.

The meeting concluded with an eloquent appeal by the Right Hon. Andrew Fisher on behalf of all seafaring men, the bravest of all and the least inclined to complain, who manifest in the highest degree the qualities of which we stand most in need, and with a tribute from Dr. Frederick Taylor to the fine efforts being made on every hand to cope with the new and trying situation created by the understaffing of hospitals on account of the large requirement of medical men in the combatant Services. In some cases men were serving in lower hospital posts than they had previously occupied in order to contribute what help they could.

